# The Added Value of Sterility in Minor Surgical Procedures in Preventing Infection: A Systematic Review

**DOI:** 10.3390/healthcare12212101

**Published:** 2024-10-22

**Authors:** Anissa Mahraoui, J. Carel Goslings, Wouter P. Kluijfhout

**Affiliations:** Department of Surgery, OLVG Hospital, Jan Tooropstraat 164, 1061 AE Amsterdam, The Netherlands; j.c.goslings@olvg.nl

**Keywords:** infection, sterility, minor surgical procedure, outpatient surgical procedure

## Abstract

**Background:** The necessity of maintaining sterility during minor surgical procedures is a debated topic due to concerns over the cost, environmental impact of sterile supplies, and the unclear benefits of sterility in minor surgical procedures. This review aims to evaluate the available evidence on this topic. **Methods:** A systematic review of studies comparing sterile and non-sterile techniques in minor surgical procedures was conducted. Databases searched included PubMed and Cochrane up to May 2024. Studies were selected based on predefined criteria. **Results:** A total of eight studies met the inclusion criteria. Non-sterility was mostly defined by the use of non-sterile gloves, whereas the remainder of the procedure was performed with the same technique as a sterile procedure. The analysis showed no significant difference in infection rates between sterile and non-sterile techniques. However, sterile techniques may reduce the risk of complications in specific contexts, such as in immunocompromised patients or in procedures performed in tissues deeper than subcutaneous fascia. **Conclusions:** The evidence suggests that for most minor surgical procedures, non-sterile techniques do not significantly increase the risk of postoperative infections. Further high-quality studies are needed to identify specific scenarios where sterility can be safely omitted to decrease surgical waste and costs without increasing the risk of infection.

## 1. Introduction

Infection prevention is a crucial aspect within healthcare, especially in environments where surgical procedures take place. Since Semmelweis discovered that surgeons with unwashed hands can transmit diseases between patients almost two centuries ago, much research has been conducted in this field [1]. Postoperative infections can lead to increased morbidity, prolonged healing times, and additional medical interventions, imposing a significant burden on both patients and healthcare systems [2]. Traditionally, the focus of this research has been on operating rooms in hospitals. Over the last few decades, however, we have seen an increase in the performance of outpatient surgical procedures. The setting of these procedures can be in the emergency department, a general practitioner’s office, or a so-called small procedure room. The benefits of performing these procedures in an outpatient setting include reduced hospital admissions, improved accessibility for patients, lower costs, and an overall higher efficiency [3].

These minor surgical procedures, such as the excision of small skin lesions and laceration repairs, are typically brief and involve limited invasiveness compared to major surgeries [4]. Consequently, there is ongoing debate within the medical community about the necessity of strict sterility protocols for these types of interventions. Sterility measures, including the use of sterile gloves, instruments, and environments, are intended to minimize the risk of postoperative infections. However, the implementation of these protocols can be resource-intensive and time-consuming, leading to questions about their necessity [5]. Additionally, healthcare in the Netherlands is responsible for approximately 7–8% of the country’s total CO_2_ emissions and accounts for 10–13% of the national GDP [6,7]. Reducing the overuse of sterility protocols in minor procedures could potentially lower medical waste and CO_2_ emissions while also contributing to cost savings [8].

Understanding whether sterility is essential in minor surgical procedures is crucial for developing guidelines. Current clinical guidelines vary regarding the necessity of sterile techniques in minor surgeries. Some guidelines advocate for the use of sterile techniques universally, while others suggest that clean (non-sterile) techniques may be adequate for certain procedures [9,10,11]. This variability often reflects a lack of consensus in the medical literature, underlining the need for a comprehensive review of existing evidence.

Although the overall literature is scarce, some studies have explored the outcomes of sterile versus non-sterile techniques in various minor surgical contexts [12,13,14,15]. The current infection rate in the existing literature for minor outpatient surgical procedures varies between 2 and 6% [12,13,14,15,16,17,18]. Interestingly, some studies have suggested that non-sterile practices in outpatient procedures may not necessarily increase the risk of infection [19,20,21,22,23,24].

This systematic review aims to evaluate the impact of sterile versus non-sterile techniques on the rates of postoperative infections in minor surgical procedures. Specifically, the objectives are to compare infection rates associated with sterile and non-sterile techniques and identify patient populations and specific procedural contexts where sterility may offer significant benefits.

By addressing these objectives, this review seeks to contribute to the optimization of surgical practices, enhancing patient outcomes while considering sustainability and the economic aspects of healthcare delivery. Ultimately, the findings will help clinicians make informed decisions about the necessity of sterility in minor surgeries.

## 2. Methods

### 2.1. Search Strategy

This systematic review was performed according to the Preferred Reporting Items for Systematic Reviews and Meta-Analyses (PRISMA) guidelines [25]. A comprehensive search was conducted in MEDLINE (PubMed) and Cochrane, comparing sterile and non-sterile techniques for minor outpatient surgical procedures. The final search was implemented on 5 May 2024. Keywords included “sterile”, “non-sterile”, “minor surgical procedures”, “outpatient surgery”, “dermatological procedures”, and “suturing”. Additional medical subject headings (MeSH) terms and title/abstract terms used in the literature search, along with results from MEDLINE and Cochrane, are reported in the Supplementary Materials (Tables S1 and S2).

### 2.2. Inclusion and Exclusion Criteria

This review included all studies performing minor surgical procedures, regardless of the setting. Thus, procedures conducted in dermatological outpatient settings or general practitioner settings were also included. The inclusion criteria comprised randomized controlled trials (RCTs), prospective cohort studies, and retrospective cohort studies that compared the incidence of postoperative infections between sterile and non-sterile techniques in minor surgical procedures. Only records published in the last 20 years (2004–2024) were included. Exclusion criteria encompassed studies not written in English, systematic reviews, letters, comments, case reports, conference abstracts, and studies on major surgical procedures or procedures performed in traditional operating rooms.

### 2.3. Quality Assesment

The quality of the included studies was evaluated using the Cochrane Risk of Bias Tool for RCTs and the Modified Newcastle Ottawa Scale for observational studies. Each study was assessed for potential sources of bias. One independent reviewer conducted the quality assessment. The risk of bias in the studies was categorized as low, moderate, or high based on the established guidelines (Supplementary Materials; Tables S3 and S4). This quality assessment was performed to ensure that the findings of this systematic review were grounded in reliable evidence.

### 2.4. Data Extraction

Titles, abstracts, and full-text articles were scanned for eligibility by the first reviewer. References of the final included articles were checked manually for additional studies.

The extracted data included the study setting, the number of patients in the sterile and non-sterile groups, and the definition of sterility. Sterility was defined by the presence or absence of disinfection, the use of sterile instruments, and whether sterile gloves, gowns, and gauze pads were employed. Procedures were classified as dermal, subcutaneous, or subfascial to ensure valid comparisons across varying levels of invasiveness. Furthermore, the overall infection rate, as well as the infection rates specific to both the sterile and non-sterile groups following the procedures, were recorded.

### 2.5. Outcome

The primary outcome of this review was the infection rate. Given the anticipated heterogeneity in study settings and the types of procedures performed, a meta-analysis was not feasible. Instead, infection rates are described as percentages, providing a clear comparison of the outcomes associated with each technique.

## 3. Results

### 3.1. Study Selection

**Figure 1:**
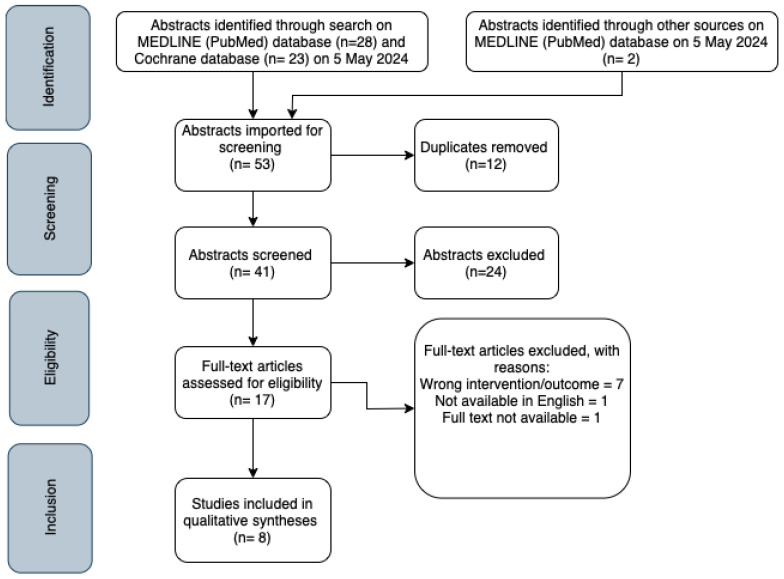
PRISMA flowchart of study selection.

The literature search in the PubMed and Cochrane databases retrieved a total of 53 abstracts. The final search was filtered to only include studies performed in the past 20 years (2004–2024). After removing duplicates, the search resulted in 41 unique abstracts. Firstly, the abstracts were screened based on title, abstract, and study design. Conference abstracts, reviews, letters, comments, and case reports were excluded. A total of 17 remaining full-text articles were assessed for eligibility. Studies were excluded if they only observed infection rates without providing data on intervention (sterile versus non-sterile procedures) or when they compared outpatient procedures to those performed in traditional operating rooms. Lastly, articles not available in full text or not written in English were also excluded. Additionally, two articles are identified through citation searches in existing articles, leading to the final inclusion of eight articles. The literature screening and reasons for exclusion are listed in Figure 1.

### 3.2. Study Characteristics

The included articles consisted of four RCTs, three prospective cohort studies, and one retrospective cohort study. The sample sizes ranged from 60 to 3491 patients, with follow-up periods varying from one week to six months [3,17,26,27,28,29,30,31].

Two studies were conducted in the emergency department (ED), one study in a general practice setting, and five studies in dermatological settings. The procedures across these studies were largely similar, primarily involving minor skin excisions for various types of skin cancer, although there was some variability in the complexity of these excisions. The excisions targeted the cutis or subcutis. As detailed in Table 1, some dermatological studies involved Mohs surgery, which also included more complex procedures with grafts, flaps, or a secondary closure. Zwaans et al. (2022) and Perelman et al. (2004) studied the added value of sterility in suturing uncomplicated lacerations (without tendon injury) in the ED [26,28].

In most studies, the primary difference between the sterile and non-sterile groups was the use of sterile versus non-sterile gloves. Other materials and instruments were used in a sterile manner as per standard practice. Only Zwaans et al. (2022) also employed non-sterile dressings, drapes, and gauzes in the non-sterile group [28]. The study characteristics are summarized in Table 1.

### 3.3. Infection Rates

Across the included studies, infection rates ranged from 0.61% to 9.0%. Generally, most studies found no significant difference in infection rates between sterile and non-sterile techniques for minor surgical procedures. However, some studies indicated that sterile techniques might offer advantages in specific high-risk groups or procedures involving deeper tissues. Rogues et al. (2007) reported a statistically significant lower infection risk with sterile gloves in reconstructive procedures involving deeper tissues, but no difference in infection risk for simpler procedures [27]. Similarly, Zwaans et al. (2022) found no overall difference in infection risk between sterile and non-sterile gloves when suturing lacerations, though they noted a higher infection risk for immunocompromised patients using non-sterile gloves [28]. The infection rates for all studies are stated in Table 2.

## 4. Discussion

The findings from this systematic review suggest that for minor surgical procedures, non-sterile techniques do not significantly increase the risk of postoperative infections or complications. Non-sterility in these studies was predominantly defined as the use of non-sterile gloves, whilst the rest of the procedure was performed as usual. The majority of included studies reported similar infection rates between sterile and non-sterile techniques, with only a few studies indicating marginally lower infection rates with sterile techniques in specific high-risk groups, such as immunocompromised patients [28].

The most important finding of this review is that infection rates between sterile and non-sterile techniques are often comparable. Across the included studies, infection rates for both sterile and non-sterile techniques were similar, ranging from 1.8% to 6.3%, with extreme outliers at 9.0% and 0.61% [3,19,26,27,28,29,30,31]. This result may be explained by the relatively superficial nature of minor surgical procedures, which typically involve limited invasiveness and reduced exposure to deeper tissues, thus decreasing the likelihood of infection. Many of the reviewed procedures, such as skin excisions and laceration repairs, involve anatomical structures with a robust vascular supply and a lower propensity for infection, which might explain why non-sterile techniques are generally safe in these contexts [32,33,34].

However, the review also identified that sterility may be beneficial in certain specific scenarios, particularly in more complex procedures or among higher-risk patient populations. For instance, procedures involving deeper tissues, such as those that extend below the muscle fascia, may warrant the use of sterile techniques to reduce infection risk [27]. Immunocompromised patients, due to their reduced ability to fight infections, may also benefit from stricter sterility measures. Rogues et al. (2007) demonstrated that sterile gloves significantly reduced infection rates in reconstructive procedures involving deeper tissue layers, while Zwaans et al. (2022) reported a higher infection risk for immunocompromised patients when non-sterile gloves were used [27,28]. These findings suggest that sterility should be selectively applied based on patient vulnerability and procedural complexity.

The wide variability in infection rates across the studies, from as low as 0.61% to as high as 9.0%, warrants further discussion. Heal et al. reported a notably higher infection rate of 9.0%, which may be attributed to their study population’s older age (mean age 65.3 years) and the study’s hot and humid climate in Mackay, both of which are known to increase infection risk [3]. Nevertheless, they concluded that the effect of sterility would likely be similar in cohorts with lower baseline infection rates, even in more temperate climates. Additionally, Zwaans et al. (2022) reported a relatively high infection rate, which can be explained by the nature of the wounds treated, which were predominantly emergency department cases [28]. These wounds were typically caused by contaminated objects rather than clean surgical instruments, suggesting that the use of non-sterile gloves may be less consequential when the wound itself is already contaminated. On the other hand, Mehta et al. reported an exceptionally low infection rate of 0.61%, which could be due to several factors. Their study excluded higher-risk cases, such as those involving extremities or the ear, where prophylactic antibiotics were administered, and cases referred for plastic surgery reconstruction, which followed a different postoperative care pathway. The exclusion of such high-risk cases could have resulted in a lower overall infection rate. Additionally, Mohs surgery was performed in a specialized center with high case volumes and strict protocols, which may have further contributed to the low infection rate. This contrasts with less experienced providers in emergency departments or general practice settings, where less rigorous protocols and lower case volumes might result in higher infection rates [30].

The variability in the use of sterile materials across studies also complicates the interpretation of results. While some studies focused exclusively on the use of sterile gloves, others extended sterility to include drapes and instruments. Notably, some studies on Mohs surgery used non-sterile gloves during only part of the procedure (tumor extraction phase), while others used them throughout the entire procedure, including reconstruction [19,29,30]. This means that except for the gloves, all other aspects of sterility, such as disinfection, sterile instruments, and sterile dressings, were consistently maintained. The only exception is the study by Zwaans et al. (2022), which involved contaminated wounds, making it unique in its context [28]. Therefore, we must be cautious in drawing broad conclusions, as the non-sterile performed procedures were tightly controlled in most studies, except in cases where the wounds themselves were already contaminated.

To the best of our knowledge, this is the first systematic review to address the comparison between sterile and non-sterile techniques in minor surgical procedures, positioning it as a timely contribution to a topic of growing relevance in clinical practice. Another strength of this review is its methodological rigor, as it was conducted in accordance with PRISMA guidelines and employed a qualitative approach to synthesize the available evidence. Nevertheless, several limitations should be considered. There was considerable heterogeneity in study sizes and infection outcomes, which may impact the overall generalizability of the results. Furthermore, the variation in patient populations, procedural characteristics, and definitions of sterility across the studies complicates direct comparisons and limits the strength of the conclusions.

From a broader perspective, the potential benefits of reducing sterility protocols in minor procedures extend beyond clinical outcomes. Healthcare systems are increasingly recognizing the economic and environmental costs of maintaining strict sterility. Sterile procedures require the use of single-use gloves, drapes, instruments, and other supplies, all of which contribute to medical waste and carbon emissions. In the Netherlands, healthcare is responsible for 7–8% of the nation’s CO_2_ emissions, and sterile materials are a substantial part of this environmental footprint [6,7,8]. By adopting non-sterile techniques where appropriate, healthcare facilities could not only reduce waste and carbon emissions but also achieve cost savings [35]. However, these potential gains must be weighed against the consequences of postoperative infections. Infections can lead to increased healthcare costs through the need for antibiotics, extended follow-up care, or even hospital admissions, which could outweigh the savings achieved by reducing sterility protocols. Therefore, clinicians should consider both the environmental and economic implications alongside patient safety when determining the necessity of sterility in minor procedures.

## 5. Conclusions

In our study, based on the analysis of eight studies, we conclude that non-sterile gloves can be safely used for most minor surgical procedures without significantly increasing the risk of postoperative infections or complications. These findings support a more flexible and context-dependent approach to sterility, with potential benefits in terms of cost savings, resource allocation, and environmental sustainability. Further research is needed to investigate whether the definition of non-sterility can be extended to more than non-sterile gloves alone, such as using non-sterile gauzes. Additional data are also needed to provide recommendations for specific patient populations and procedural contexts. By integrating clinical efficacy with economic and environmental considerations, healthcare providers can enhance patient care while promoting a more sustainable and efficient healthcare system.

## Figures and Tables

**Table 1:** Study characteristics.

	Design	Procedures	Setting	Sterility	Total (N)	Sterile (N)	Non-Sterile (N)
Michener et al. (2019) [31]	RCT	Simple skin excisions	DM	Gloves	106	53	53
Perelman et al. (2004) [26]	RCT	Suturing of lacerations	ED	Gloves	816	408	408
Xia et al. (2011) [29]	RCT	Mohs surgery	DM	Gloves	60	30	30
Zwaans et al. (2022) [28]	RCT	Suturing of lacerations	ED	Gloves, dressing, drapes, gauzes	1480	747	733
Heal et al. (2015) [3]	Prospective	Simple skin excisions	GP	Gloves	493	243	250
Mehta et al. (2014) [30]	Prospective	Mohs surgery	DM	Gloves	1883	942	941
Rogues et al. (2007) [27]	Prospective	Simple and reconstructive skin excisions	DM	Gloves	3491	2582	909
Rhinehart et al. (2006) [19]	Retrospective	Mohs surgery	DM	Gloves	1239	560	679

GP: general practice, DM: dermatology procedure rooms/private office, ED: emergency department.

**Table 2:** Infection rates.

	Total Infection (%)	Sterile Infection (%)	Non-Sterile Infection (%)	Conclusion
Michener et al. (2019) [31]	2.9	3.8	1.9	No difference
Perelman et al. (2004) [26]	5.3	6.1	4.4	No difference
Xia et al. (2011) [29]	5	6.6	3.3	No difference
Zwaans et al. (2022) [28]	6.3	6.8	5.7	No difference
Heal et al. (2015) [3]	9.0	9.3	8.7	No difference
Mehta et al. (2014) [30]	0.61	0.56	0.65	No difference
Rogues et al. (2007) [27]	1.9	Simple procedures: 1.6	Simple procedures: 1.7	No difference for simple excisions
	9.1	Reconstructive procedures: 3.4	Reconstructive procedures: 14.7	Lower risk for infection with sterile gloves in reconstructive procedures
Rhinehart et al. (2006) [19]	1.8	1.7	1.8	No difference

## Data Availability

Not applicable.

## Supplementary Materials

The following supporting information can be downloaded at: https://www.mdpi.com/article/10.3390/healthcare12212101/s1, Table S1: Search in MEDLINE (Pubmed) database (5 May 2024); Table S2: Search in Cochrane Library database (5 May 2024); Table S3: Risk of Bias of RCTs; Table S4: Risk of Bias of observational studies.
