# Supplementary material for: The Added Value of Sterility in Minor Surgical Procedures in Preventing Infection: A Systematic Review
Source: Healthcare (Basel). 2024 Oct 22;12(21):2101. doi: 10.3390/healthcare12212101 (PMC11545193; doi:10.3390/healthcare12212101)
Supplement: Supplementary file 1 [file healthcare-12-02101-s001.zip › healthcare-3221383-supplementary.pdf]

**Supplementary materials**

**Table S1: Search in MEDLINE (Pubmed) database (5 May 2024)**

| ID | Search terms | Hits |
|---|---|---|
| #1 | (minor surgical procedure[MeSH Terms]) OR (minor surgical procedures[MeSH Terms]) OR (minor surgery[Title/Abstract]) OR (minor surgeries[Title/Abstract]) OR (outpatient surgeries[Title/Abstract]) OR (outpatient procedure[Title/Abstract]) OR (minor surgical procedure[Title/Abstract]) OR (dermatological excision[Title/Abstract]) OR (excision[Title/Abstract]) OR (suture[Title/Abstract]) OR (suturing[Title/Abstract]) | 267,016 |
| #2 | (sterile[Title/Abstract]) OR (sterilised[Title/Abstract]) OR (sterilized[Title/Abstract]) OR (sterilization[Title/Abstract]) OR (sterile gloves[Title/Abstract]) | 87,337 |
| #3 | (nonsterile[Title/Abstract]) OR (non-sterile[Title/Abstract]) OR (non-sterile gloves[Title/Abstract]) | 2,562 |
| #4 | #1 AND #2 AND #3 | 28 |

**Table S2: Search in Cochrane Library database (5 May 2024)**

| ID | Search terms | Hits |
|---|---|---|
| #1 | MeSH descriptor: [Minor Surgical Procedures] explode all trees | 136 |
| #2 | "minor surgical procedures" OR "small surgical procedures" OR "minor surgery" OR "outpatient surgery" OR "ambulatory surgery" OR "skin biopsy" OR "skin excision" OR "suturing" OR #1 | 6915 |
| #3 | "sterile technique" OR "sterile gloves" OR "sterile field" OR "sterile procedure" OR "sterile" OR "sterility" | 14273 |
| #4 | "non-sterile technique" OR "clean technique" OR "non-sterile gloves" OR "non-sterile procedure" OR "non-sterile" OR "sterility" | 5980 |
| #5 | #2 AND #3 AND #4 | 23 |

**Table S3. Risk of bias of RCTs (Cochrane Risk of Bias Tool)**

| | Random sequence generation | Allocation concealment | Blinding of participants and personnel | Blinding of outcome assessment | Incomplete outcome data | Selective reporting |
|---|---|---|---|---|---|---|
| Michener et al. (2019) [31] | + | ? | - | ? | + | ? |
| Perelman et al. (2004) [26] | + | + | + | + | + | + |
| Xia et al. (2011) [29] | + | + | ? | + | + | + |
| Zwaans et al. (2022) [28] | + | ? | - | ? | - | ? |

+ : low risk of bias, - : high risk of bias, ? : unknown

**Table S4. Risk of bias of observational studies (Modified Newcastle Ottawa Scale)**

| | Selection of cohorts/patients | Adjusting for confounders/ comparability of cohorts | Assessment of outcome | Follow-up period | Adequacy to follow up | Source of funding reported |
|---|---|---|---|---|---|---|
| Heal et al. (2015) [3] | + | - | + | + | + | ? |
| Mehta et al. (2014) [30] | - | - | + | - | ? | ? |
| Rogues et al. (2007) [27] | + | + | + | ? | ? | ? |
| Rhinehart et al. (2006) [19] | + | - | + | + | ? | ? |

+ : low risk of bias, - : high risk of bias, ? : unknown
